# Akt1 and Akt3 but not Akt2 through interaction with DNA-PKcs stimulate proliferation and post-irradiation cell survival of K-RAS-mutated cancer cells

**DOI:** 10.1038/cddiscovery.2017.72

**Published:** 2017-10-30

**Authors:** Mahmoud Toulany, Julia Maier, Mari Iida, Simone Rebholz, Marina Holler, Astrid Grottke, Manfred Jüker, Deric L Wheeler, Ulrich Rothbauer, H Peter Rodemann

**Affiliations:** 1 Division of Radiobiology and Molecular Environmental Research, Department of Radiation Oncology, University of Tuebingen, Tuebingen, Germany; 2 German Cancer Consortium (DKTK), Partner Site Tuebingen, and German Cancer Research Center (DKFZ), Heidelberg, Germany; 3 Natural and Medical Sciences Institute at the University of Tuebingen, Reutlingen, Germany; 4 Pharmaceutical Biotechnology, Eberhard Karls University Tuebingen, Tuebingen, Germany; 5 Department of Human Oncology, University of Wisconsin, Madison, WI, USA; 6 Institute of Biochemistry and Signal Transduction, University Medical Center Hamburg-Eppendorf, Hamburg, Germany

## Abstract

Akt1 through the C-terminal domain interacts with the DNA-dependent protein kinase catalytic subunit (DNA-PKcs) and stimulates the repair of DNA double-strand breaks (DSBs) in K-RAS-mutated (K-RASmut) cells. We investigated the interactions of distinct domain(s) of DNA-PKcs in binding to full-length Akt1. Similarly, we analyzed potential interactions of DNA-PKcs with Akt2 and Akt3. Finally the effect of Akt isoforms in cell proliferation and tumor growth was tested. We demonstrated that Akt1 preferentially binds to the N-terminal domain of DNA-PKcs using pull-down studies with distinct eGFP-tagged DNA-PKcs fragments that were expressed by plasmids in combination with mCherry-tagged full-length Akt isoforms. These binding studies also indicated an interaction with the intermediate and C-terminal domains of DNA-PKcs. In contrast, Akt3 interacted with all four DNA-PKcs fragments without a marked preference for any specific domain. Notably, we could not see binding of Akt2 to any of the tested DNA-PKcs fragments. In subsequent studies, we demonstrated that Akt inhibition interferes with binding of Akt1 to the N-terminal domain of DNA-PKcs. This indicated a correlation between Akt1 activity and the Akt1/DNA-PKcs complex formation. Finally, knockdown studies revealed that the depletion of endogenous Akt1 and Akt3, but not Akt2, inhibit clonogenic activity and repair of ionizing radiation (IR)-induced DNA DSBs, leading to radiosensitization. Furthermore, in a xenograft study the expression of shAkt1 or shAkt3, but not shAkt2 in K-RASmut breast cancer cell line MDA-MB-231 showed major tumor growth delay. Together, these data indicate that Akt1 and Akt3, but not Akt2, physically interact with DNA-PKcs, thus stimulating the repair of DSBs and therefore protecting K-RASmut cells against IR. Likewise, interaction of Akt isoforms with DNA-PKcs could be crucial for their role in regulating tumor growth.

## Introduction

The major mechanisms that lead to a constitutive activation of the PI3K/Akt pathway are mutations and overexpression of upstream receptor tyrosine kinases such as erbB family members, activating mutations of PIK3CA or RAS and the loss of tumor suppressor protein phosphatase and tensin homolog (PTEN).^1^ Akt, also known as protein kinase B (PKB), consists of three isoforms: PKBα/Akt1, PKBβ/Akt2 and PKBγ/Akt3. Akt isoforms have a N-terminal PH (pleckstrin homology) domain and a kinase domain, which are separated by a 39-amino-acid hinge region.^2^ The PH domains are approx. 60% identical and the kinase domains are more than 85% identical.^3^ Catalytically active Akt regulates the function of numerous substrates involved in cell survival, growth, proliferation, metabolism and protein synthesis (reviewed in Manning, Cantley^4^).

K-RAS mutated in codon 12 as well as in codon 13 stimulates autocrine production of EGFR ligands and enhances basal activation of the PI3K/Akt pathway.^5,6^ Likewise, K-RAS mutation leads to enhanced cell proliferation and tumor cell clonogenicity.^6^ Akt1 was implicated in the repair of radiation-induced DNA damage in K-RAS-mutated cells.^6,7^ Previous studies including our own demonstrated that after irradiation, a physical interaction of Akt1 is induced through its C-terminal domain with the catalytic subunit of DNA-dependent protein kinase (DNA-PKcs).^8,9^ Through this interaction Akt1 promotes the kinase activity and autophosphorylation of DNA-PKcs,^8,10–12^ as a core enzyme involved in repair of DNA double-strand breaks (DSBs) through non-homologous end joining (NHEJ),^8,11,13^ and the release of DNA-PKcs from the damage site.^8^ Thus, Akt1 can be considered as a kinase that is involved in NHEJ of DSBs and radioresistance.^8,11,13,14^

The activation of DNA-PKcs by Akt1 in K-RAS-mutated cells may be dependent on the binding of Akt1 to a specific domain of DNA-PKcs. Thus, we analyzed the interaction of Akt1 and DNA-PKcs in more detail. We performed pull-down studies to identify the individual domains of DNA-PKcs that bind to full-length Akt1 in K-RAS-mutated NSCLC cells. Additionally, we expanded our binding analysis to full-length Akt2 and Akt3 to investigate whether the other Akt isoforms interact in a comparable manner with DNA-PKcs in NSCLC as well as in breast cancer cells. Likewise, we investigated the function of different Akt isoforms in the process of DNA repair. The data indicate that Akt1 and Akt3, but not Akt2, interact with DNA-PKcs. Consistent with this observation, knockdown of endogenous Akt1 and Akt3 impaired the repair of radiation-induced DSBs and promoted radiosensitization, but this effect was not observed by Akt2 knockdown. Similarly, knockdown of Akt1 and Akt3 significantly hampered cell proliferation *in vitro* and tumor growth *in vivo*, but Akt2 knockdown did not.

## Results

### Overexpression of Akt isoforms and DNA-PKcs fragments in A549 and HEK293T cells

DNA-PKcs physically interacts with the C-terminal domain of Akt1.^8,15^ It is still unclear, however, whether the other isoforms of the Akt family (Akt2, Akt3) also interact with DNA-PKcs in a similar way and which domain of DNA-PKcs constitutes the binding site that is recognized by the different Akt isoforms.

**Figure 1:**
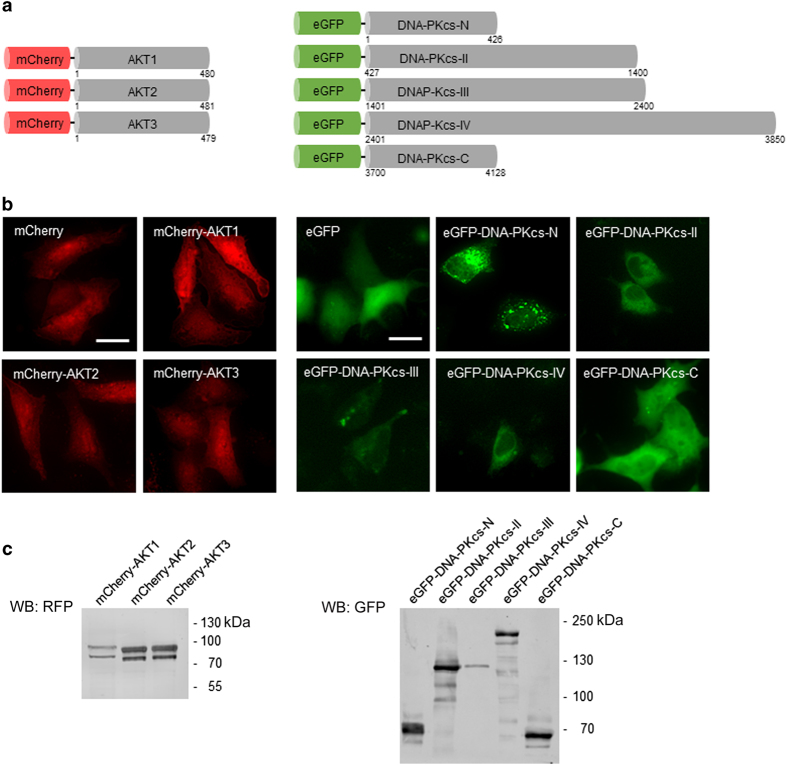
Overexpression of Akt isoforms and DNA-PKcs in A549 or HEK293T cells. Expression of Akt and DNA-PKcs fusion proteins. (**a**) Schematic overview of AKT1, AKT2 and AKT3 constructs fused to mCherry as well as the constructs expressing different subdomains of DNA-PKs fused to eGFP. (**b**) A549 cells were transfected with the indicated constructs. Twenty-four hours after the transfection, fluorescent images were acquired. Cells expressing mCherry or eGFP alone served as controls. Scale bars: 20 μm. (**c**) HEK293T cells were transfected with the indicated constructs. Twenty-four hours after the transfection, the cells were lysed and eGFP fusion proteins were precipitated using the GFP-Trap. Whole-cell lysates (mCherry) and the bound fractions (eGFP) were subjected to SDS-PAGE followed by western blot analysis using antibodies specific for mCherry or eGFP.

For the biochemical analysis of cellular expressed proteins, we generated expression constructs by fusing the N-termini of Akt1, Akt2 or Akt3 to mCherry (Figure 1a). In parallel, we constructed a series of five DNA-PKcs mapping regions (-N, a.a. 1–426; -II, 427–1400; -III, 1401–2400; -IV, 2401–3850; -C, 3700–4128) and generated N-terminal eGFP fusions (Figure 1a). To examine the cellular expression of the corresponding fusion proteins, we transiently transfected A549 and HEK293T cells with the respective expression constructs and performed fluorescence imaging (Figure 1b) and immunoblotting of whole-cell lysates (Figure 1c). We used antibodies directed either against mCherry (Figure 1c, left panel) or eGFP (Figure 1c, right panel).

Whereas all the Akt constructs showed comparable expression levels and a diffuse cellular distribution, we observed differences in expression level as well as intracellular distribution for DNA-PKcs constructs. Thus, eGFP-DNA-PKcs-N was diffusely distributed throughout the cells, while eGFP-DNA-PKcs-II, -III and -C were exclusively localized in the cytoplasm. Notably only eGFP-DNA-PKcs-IV showed a slight enrichment in the nucleus (Figure 1b). Two bands were detectable for every construct in the immunoblot analysis of the mCherry-tagged Akt proteins using either anti-mCherry or gene-specific antibodies (Figure 1c, left panel). Further analysis indicated that the band corresponding to the lower molecular weight represented a full-length Akt protein with a protease-processed form of the mCherry protein (data not shown). Although this cleavage was slightly reduced at lower temperatures or by addition of higher concentrations of protease inhibitors, it could not be completely avoided. The results of the western blot of the eGFP-tagged domains of DNA-PKcs showed that all constructs were correctly expressed and detectable at the expected sizes (Figure 1c, right panel). Due to the similar expression of the plasmids covering N- and C-terminal domains of DNA-PKcs in HEK239 cells (Figure 1c), those constructs were applied to analyze the potential binding of the Akt isoforms to the N- and C-terminal domain of DNA-PKcs.

### Akt1 and Akt3 but not Ak2 interact with DNA-PKcs

**Figure 2:**
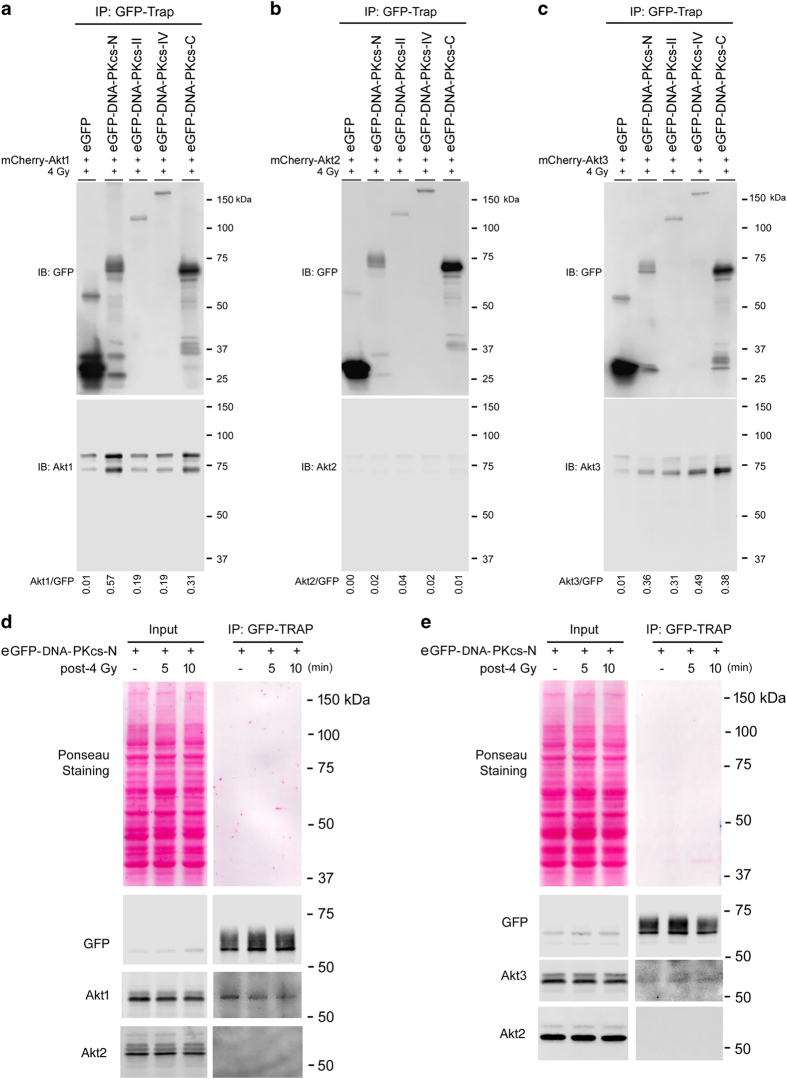
Akt1 and Akt3 but not Ak2 interact with DNA-PKcs. K-RAS-mutated A549 cells were transfected with the indicated plasmids. Forty-eight hours after the transfection, cells were irradiated with 4 Gy and lysed 10 min post-irradiation. IP of eGFP was performed using GFP-Trap. The co-IPs for Akt1 (**a**) Akt2 (**b**) and Akt3 (**c**) were analyzed by western blotting using Akt-specific antibodies. The densitometry values represent the ratios of Akt1/eGFP (**a**), Akt2/eGFP (**b**) and Akt3/eGFP (**c**). Cells were transfected with eGFP-DNA-PKcs-N and were either mock irradiated or irradiated with 4 Gy. Subsequently, the cells were lysed at the indicated times post-irradiation and the eGFP-tagged proteins were precipitated from the soluble protein fraction. The bound fractions were subjected to SDS-PAGE and immunoblot analysis using anti-eGFP (**d**, **e**), antibodies against the Akt1 and Ak2 isoforms (**d**) or against the Akt2 and Akt3 isoforms (**e**). Ponseau staining was shown for locating protein bands on immunoblots.

A549 cells were transfected with the expression vectors that coded for the various mCherry-tagged Akt isoforms in combination with expression constructs that coded for only eGFP or the eGFP-tagged DNA-PKcs fragments. Forty-eight hours after transfection, cells were irradiated with 4 Gy and the soluble protein fractions were collected 10 min later. Immunoprecipitation (IP) was performed by incubating the soluble protein fractions with the GFP-Trap. Subsequently, the bound fractions were subjected to SDS-PAGE and immunoblotting analysis. The antibody detection revealed a strong signal of precipitated eGFP in the bound fraction (IP) of cells that expressed isolated eGFP and the corresponding mCherry-tagged Akt isoforms. Due to the strong enrichment of eGFP, we observed a small fraction of co-precipitated Akt1 or Akt3. Precipitation of the eGFP-labeled fragments of DNA-PKcs led to a clear enrichment of mCherry-tagged Akt1 in the bound fraction of eGFP-DNA-PKcs-N and eGFP-DNA-PKcs-C. We only detected minor signals, however, for Akt1 upon the precipitation of eGFP-DNA-PKcs-II and -IV (Figure 2a).

Interestingly, we did not observe any Akt2 binding upon co-expression and precipitation of eGFP-DNA-PKcs fragments (Figure 2b). In contrast, Akt3 showed a strong binding to the C-terminal domain of DNA-PKs; however, it also co-precipitated with the other fragments to a lower extent (Figure 2c). Lack of binding of Akt2 to DNA-PKcs could be due to a lower level of expression of Akt2 compared with the expression level of Akt1 and of Akt3. Thus, to rule out this possibility, the interaction of each of the three Akt isoforms in one set of experiments was tested after co-transfecting cells either with mCherry-Akt1, -Akt2 or -Akt3 followed by mock irradiation or irradiation with 4 Gy. The data presented in Supplementary Figure S1 indicate that mCherry-Akt1 and -Akt3 but not mCherry-Akt2 interacted with DNA-PKcs following mock irradiation and under irradiated conditions. Analysis of the expression of Akt isoforms from the total lysates as input (input, Supplementary Figure S1) suggest that the expression of all three isoforms was actually similar. (Supplementary Figure S1). Similarly, to the data presented in Figures 2a–c, co-IP with the C-terminal domain of DNA-PKcs was observed with Akt1 and Akt3, but was not for Akt2 (Supplementary Figure S1). In an additional experiment, we tested the expression patterns of the eGFP-tagged Akt isoforms and compared them with the expression levels of the endogenous Akt isoforms in cells co-transfected with mCherry-DNA-PKcs-N following mock irradiation or irradiation with 4 Gy. The data from this experiment showed that the expression level of GFP-Akt2 was similar to Akt1 and higher than the expression of eGFP-Akt3 (Supplementary Figure S2). Together, these sets of data suggest a differential binding behavior of the Akt isoforms to DNA-PKcs. Thus, a lack of binding of Akt2 does not seem consequential for differential expression of Akt isoforms following transfection.

In addition, we investigated the binding of the eGFP-DNA-PKcs-N domain to the endogenous Akt isoforms (Figure 2d) 5 and 10 min post-irradiation. We performed IP as described, and we analyzed bound fractions of eGFP-DNA-PKcs using anti-eGFP or gene-specific Akt antibodies. These experiments confirmed the results previously collected of Akt1 and DNA-Pkcs interaction (Figures 2a–c, Supplementary Figure S1). After long-term exposure, a faint band could be observed for Akt3 in the bound fraction (Figure 2e), whereas no band for Akt2 was detected (Figures 2d–e).

### Targeting Akt inhibits Akt1/DNA-PKcs complex formation

**Figure 3:**
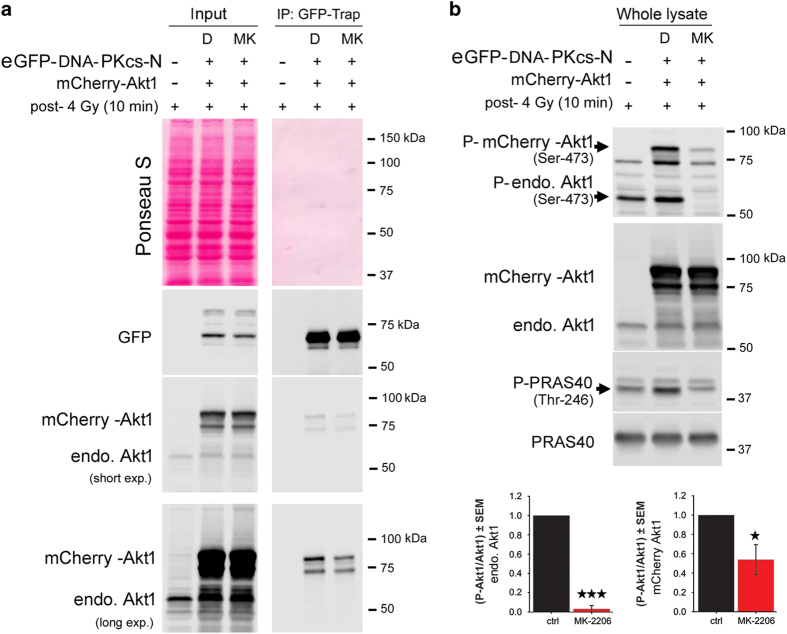
Targeting Akt inhibits Akt/DNA-PKcs complex formation. K-RAS-mutated A549 cells were transfected with eGFP-PKcs (a.a. 1-421) and mCherry-Akt1. (**a**) Forty-eight hours after transfection, the cells were treated with DMSO (D) or 10 *μ*M of MK2206 (MK) for 1 h and then irradiated with 4 Gy. The cells were lysed 10 min post-IR and eGFP-DNA-PKcs-N was precipitated as described. The input and bound fractions were subjected to SDS-PAGE and immunoblot analysis, and eGFP was detected in the inputs and under the IP conditions as the loading control. The experiment was performed in two biological replicates. The results from one experiment are shown. (**b**) The cells were lysed 10 min past-IR and immunoblot analysis was subjected. The phosphorylation of Akt (Ser-473) and PRAS40 (Thr-246) was analyzed by immunoblotting in the whole-cell lysates using phospho-specific antibodies. The blots were stripped and re-probed with the antibodies against Akt1 and PRAS40. Data indicates mean P-Akt (Ser-473)±S.E.M. from three independent experiments. The asterisks indicate a significant inhibition of Akt phosphorylation following pretreatment with MK2206 (5 *μ*M) for 2 h followed by irradiation with 4 Gy (*P<0.05, ***P<0.001, Student's *t*-test).

Next, we asked whether the observed interaction depends on Akt activity. A549 cells transiently transfected with mCherry-labeled Akt1 and eGFP-DNA-PKcs-N were treated with allosteric Akt inhibitor MK2206 (MK) 5 *μ*M for 1 h and irradiated with 4 Gy. Subsequently, cells were lysed 10 min after irradiation, and the soluble protein fraction was subjected to IP using the GFP-Trap. The input and bound fraction of the Co-IP were analyzed using anti-eGFP antibody and Akt1 antibody to detect both endogenous Akt1 and mCherry-tagged Akt1. The results showed that pretreatment with MK led to an approximately 40% reduction in binding of Akt1 to DNA-PKcs-N (Figure 3a). This minor inhibitory effect of the Akt inhibitor on complex formation of mCherry-Akt1 with eGFP-DNA-PKcs-N may be due to the lack of effect of the inhibitor on the activation of mCherry-tagged Akt1. We confirmed this hypothesis by determining the phosphorylation of endogenous Akt1 and mCherry-tagged Akt1 at Ser-473. MK inhibited phosphorylation of endogenous Akt at Ser-473 by approximately 90% while the inhibitory effect on phosphorylation of mCherry-Akt1 was only around 50% (Figure 3b). As supported by the data presented in Figure 3a, the inhibition of the complex formation of mCherry-Akt1 and eGFP-DNA-PKcs-N (Figure 3a) was correlated with the level of inhibition of phosphorylation of mCherry-Akt1 but not with the phosphorylation/activation of endogenous Akt.

### Akt1 and Akt3 but not Akt2 stimulate IR-induced DSBs

**Figure 4:**
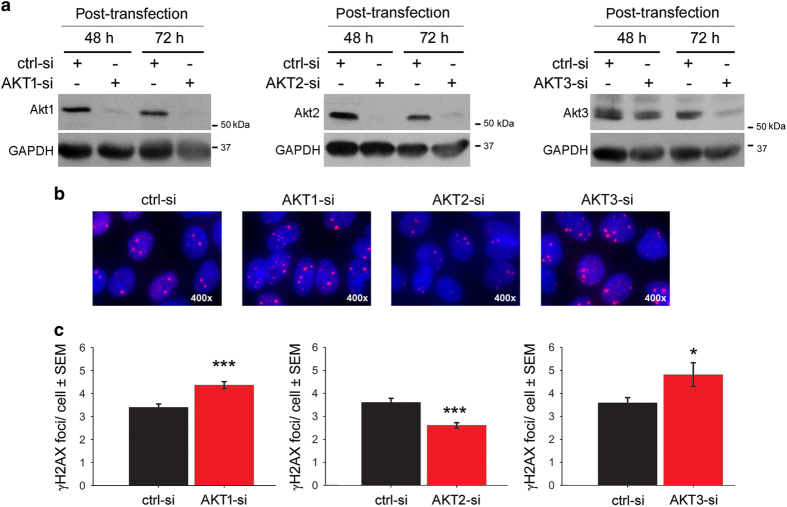
Akt1 and Akt3 but not Akt2 stimulate repair of IR-induced DSBs. (**a**) A549 cells were grown on glass slides and transfected with 50 nM of control-siRNA (ctrl-si), AKT1-siRNA (AKT1-si), AKT2-siRNA (AKT2-si) or AKT3-siRNA (AKT3-si). Forty-eight hours to 72 hours after the transfection, protein samples were isolated and the knockdown efficiency was tested by immunoblotting. In parallel to protein isolation, additional cells were irradiated with 4 Gy, and 24 h after irradiation the γ-H2AX foci were analyzed as described in Materials and Methods. (**b**) Representative image for the residual DSBs in cells transfected with control-siRNA and siRNA against different Akt isoforms 24 h after irradiation with 4 Gy. (**c**) Figures are presenting the mean numbers of foci/cell±S.E.M. from four independent replicates (800 nuclei) of the AKT1-siRNA experiment, three independent replicates (600 nuclei) of the AKT2-siRNA experiment and two independent replicates (400 nuclei) using the Akt3-siRNA (*P<0.05; ***P<0.001).

We examined the number of residual γH2AX foci to determine whether the interaction of Akt1 or Akt3 with DNA-PKcs has a functional effect on the repair of DNA DSBs. Compared with the non-target-siRNA-transfected cells, knockdown of the endogenous Akt1 or Akt3 (Figure 4a) led to a significant increase in the residual γH2AX foci 24 h after irradiation as demonstrated by the images (Figure 4b) and the statistical analyses (Figure 4c). Knockdown of Akt2 significantly reduced the number residual γH2AX foci at 24 h post-irradiation, which may imply that Akt2 is blocking DNA DSBs repair.

### Effect of Akt isoforms and DNA-PKcs on post-irradiation cell survival of K-RAS-mutated cells

**Figure 5:**
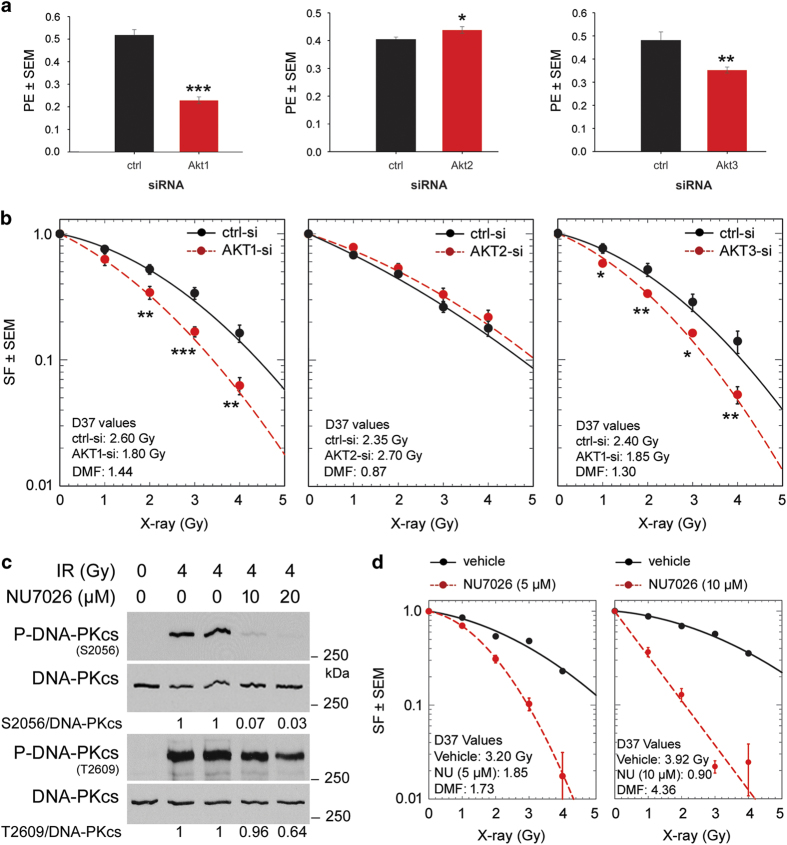
Effect of Akt isoforms and DNA-PKcs on post-irradiation cell survival of K-RAS-mutated A549 cells. (**a**) Forty-eight hours after the transfections with the indicated siRNAs, the cells were plated in six-well plates, the colonies were stained after about 10 days and the plating efficiencies were calculated by dividing the number of colonies formed to the number of cells seeded. The data presented are the mean plating efficiencies (PE)±S.E.M. of 12 replicates from two independent experiments. (**b**) Transfected cells with indicated siRNA were plated and X-ray irradiated 24 h later and then incubated for 10 days. Thereafter, the colonies were stained, and the survival fractions (SF) were calculated as described in the Materials and Methods section. The data presented are the mean survival fraction±S.E.M. of 12 replicates from two independent experiments. (**c**) Confluent A549 cells were treated with the vehicle (DMSO) or the DNA-PKcs inhibitor NU7026 at indicated concentrations for 1 h and then irradiated with 4 Gy. Protein samples were isolated 30 min after irradiation, and levels of P-DNA-PKcs (Ser-2056) and P-DNA-PKcs (Thr-2609) were determined by immunoblotting. The blots were then stripped and incubated with the DNA-PKcs antibody. (**d**) A549 cells were plated in six-well plates and 24 h later were treated with the vehicle (DMSO) or the indicated concentrations of the DNA-PKcs inhibitor NU7026 for 1 h. The cultures were then irradiated and incubated for 10 days. Thereafter, the colonies were stained, and the clonogenic fractions were calculated as described in Materials and Methods section. The data presented are the mean survival fraction±S.E.M. of six replicates from the parallel experiments. The asterisks indicate a statistically significant inhibition of plating efficiency (**a**) and radiosensitization after knockdown of Akt1 or Akt3 (**b**) (*P<0.05; **P<0.01; ***P<0.001).

Residual DNA DSBs are the major cause of cell death and clonogenic inactivation induced by IR. Thus, we investigated whether knockdown of Akt isoforms differentially affects clonogenic activity alone as well as in combination with IR. Data presented in Figure 5a demonstrate that knockdown of Akt1 and Akt3 blocked clonogenic activity significantly in non-irradiated A549 cells. In contrast, knockdown of Akt2 improved clonogenic activity under non-irradiated condition. Furthermore, combination of IR with AKT1-siRNA or AKT3-siRNA but not of AKT2-siRNA led to radiosensitization by dose modification factor (DMF) of 1.44 and 1.30, respectively (Figure 5b). Interestingly, knockdown of Akt2 led to a slight radioprotection that is reflected by a DMF of 0.87 (Figure 5b), which goes along with repair foci numbers (Figure 4c).

DNA-PKcs kinase activity and its phosphorylation are partially regulated by the Akts.^8,10–12^ Likewise, Akt-mediated repair of DNA DSBs^11^ and post-irradiation cell survival is DNA-PKcs-dependent.^8^ We investigated whether the radiosensitizing effect obtained by DNA-PKcs inhibition is comparable to the effect by targeting of each Akt isoform. In addition to the autophosphorylation sites, DNA-PKcs need to be transphosphorylated by other kinases such as ATM.^16^ We performed an experiment to identify the concentration of the DNA-PKcs inhibitor NU7026 to analyze the functional role of Akt isoforms in radiosensitization (Figure 5c). The results indicated that NU7026 inhibited the autophosphorylation of DNA-PKcs at Ser-2056 from 10 *μ*M while having a minimum effect on its transphosphorylation at Thr-2609. DNA-PKcs inhibitor NU7026 at a concentration of 10 *μ*M inhibited S2056 phosphorylation by approximately 93%, whereas the phosphorylation of T2609, a transphosphorylation site regulated by ATM, was reduced by only 4%. Increasing the concentration of NU7026 by a factor of 2 (20 *μ*M) improved the inhibition of S2056 by only 4% while the inhibition of T2609 was enhanced by approximately 32% (Figure 5c). This may be due to an off-target effect of the DNA-PKcs inhibitor NU7026 on ATM kinase activity. Based on these experiments, we tested the radiosensitizing effect of NU7026 at 10 *μ*M and below (5 *μ*M) in A549 cells. The data revealed a concentration-dependent radiosensitization by NU7026, as shown by the DMF of 1.73 at 5 *μ*M of the inhibitor and DMF of 4.36 at 10 *μ*M of the inhibitor. These data suggest that inhibition of DNA-PKcs phosphorylation leads to a markedly stronger radiosensitizing effect than knockdown of Akt1 or Akt3 (Figure 5d).

### Knockdown of Akt1 and Akt3 but not Akt2 inhibits proliferation and tumor growth in K-RAS-mutated breast cancer cells

K-RAS mutation in codon 12 or in codon 13 stimulates autocrine production of EGFR ligands and enhances basal activity of the PI3K/Akt pathway.^5,6^ Likewise, K-RAS mutation leads to stimulated proliferation as well as clonogenicity of tumor cells.^6^ Furthermore, K-RAS mutation results in enhanced Akt activity, an Akt-dependent increase in radiation-induced phosphorylation of DNA-PKcs, and consequently a stimulated repair of radiation-induced DNA DSBs, leading to radioresistance.^17^ In addition to its classical role in repair of DNA DSBs through NHEJ, DNA-PKcs affects multiple tumor-associated pathways, regulates metabolism and functions as a transcription factor (reviewed in Goodwin *et al.*^18^). Therefore, we utilized breast cancer cell line MDA-MB-231, which expresses K-RAS mutation^19^ to test if there is any correlation between binding of Akt isoforms to DNA-PKcs and their function in regulating cell proliferation *in vitro* and tumor growth *in vivo*.

We observed similar binding properties of Akt isoforms with DNA-PKcs as previously detected in A549 cells in parental K-RAS-mutated MDA-MB-231 cells (Supplementary Figure S3). Thus, in cells co-transfected with either of the Akt isoforms and the eGFP-DNA-PKcs-N a complex formation of Akt1 and Akt3 but not of Akt2 with DNA-PKcs was detectable (Supplementary Figure S3).

**Figure 6:**
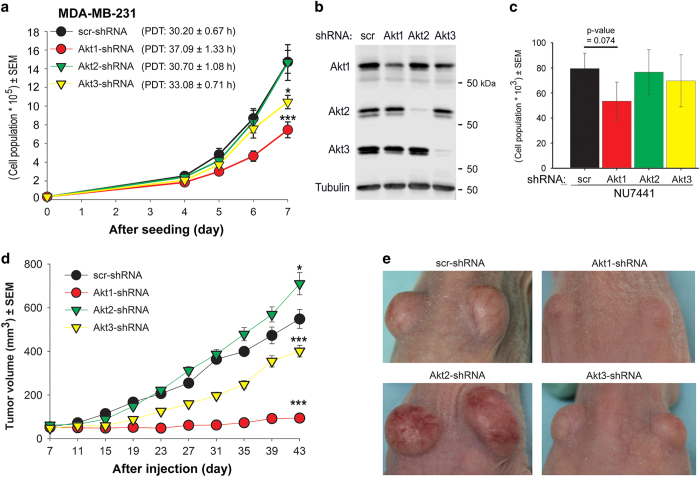
Knockdown of Akt1 and Akt3 but not Akt2 inhibits proliferation and tumor growth in K-RAS-mutated MDA-MB-231 cells. (**a**) Cells (3×10^4^) were plated in 6 cm culture dishes. At the indicated days after seeding, cells were counted and graphed. The data points represent the mean cell counts±S.E.M. of eight parallel experiments from two independent experiments. Asterisks indicate significant prolongation of PDT after knockdown of Akt1 and Akt3 compared with scramble-shRNA (scr-shRNA) (*P<0.05, ***P<0.001). (**b**) Protein samples were isolated from the cells counted on day 7 and expression of Akt isoforms was tested by immunoblotting. (**c**) Indicated cells (3×10^4^) were plated for 24 h and treated with DNA-PKcs inhibitor NU7441 (10 *μ*M). Cells were count on day 6 after treatment and graphed. Data present mean cells numbers of eight data±S.E.M. obtained from two independent experiments. (**d**) Nude mice were injected with indicates cells (2×10^6^ cells) in both dorsal flank and tumor growth assay was performed as described in Materials and Methods section. Data present mean tumor volume±S.E.M. of 14 tumors (seven mice) inoculated with MDA-MB-231-expressing scr-shRNA and of 12 tumors from six animals inoculated with MDA-MB-231 cells expressing Akt1-, Akt2- or Akt3-shRNA. Asterisks indicate a significant tumor growth delay by knockdown of Akt1 as well as Akt3 (***P<0.001) and increased in tumor volume by knockdown of Akt2 (*P<0.05), measured 6 weeks after inoculation. (**e**) Representative images of tumors following inoculation of MDA-MB-231 cells expressing scr-shRNA as well as shRNA against the Akt isoforms.

Next, we determined whether cell proliferation and tumor growth of K-RAS-mutated cells can be differentially affected by different Akt isoforms. This study was performed in MDA-MB-231 cells presenting stable knockdown of different Akt isoforms. Based on an *in vitro* proliferation assay using these cells, we showed that knockdown of Akt1 strongly inhibits cell proliferation when compared with control cells expressing scramble-shRNA (Figure 6a). This is reflected by a significant prolongation of population doubling time (PDT) (*P*<0.001) from 30.20±0.67 h in scramble-shRNA-expressing cells to 37.09±1.33 h in Akt1 knockdown cells. Knockdown of Akt3 also led to a significant (*P*<0.05) prolongation of PDT to 33.08±0.71 h. Akt2-shRNA did not affect cell proliferation as reflected by PDT of 30.70±1.80 (Figure 6a). Western blot data using protein samples isolated from cells on day 7 of proliferation confirmed the knockdown of Akt isoforms (Figure 6b).

Following the sixth day of treatment, we performed a proliferation assay in cells treated with DNA-PKcs inhibitor (NU7441, 10 *μ*M) to test whether the differential effect of Akt isoforms on cell proliferation is linked to the differential interaction of the isoforms with DNA-PKcs. Compared with cells not treated with the inhibitor (Figure 6a), NU7441 inhibited the proliferation of cells expressing scr-shRNA or shRNAs against Akt isoforms (Figure 6c). In the presence of the inhibitor, Akt1-shRNA slightly reduced cell proliferation; however, this effect was not significant when compared with scr-shRNA-expressing cells. Akt2-shRNA remained unchanged in cells treated with DNA-PKcs inhibitor, while the antiproliferative effect of Akt3-shRNA was abrogated (Figure 6c).

Lastly, to examine the effects of the three isoforms of Akt, we inoculated MDA-MB-231 cells expressing shRNA against the different Akt isoforms *in vivo*. As shown in Figure 6d and the representative images shown in Figure 6e, knockdown of Akt1 and Akt3 strongly inhibited tumor growth (*P*<0.001). In contrast to the effect of Akt1- and Akt3-knockdown, Akt2-knockdown significantly stimulated tumor growth.

## Discussion

Our results indicate that Akt1 preferentially binds to the N-terminal domain of DNA-PKcs. Although Akt3 showed binding affinity to DNA-PKcs as well, the specific binding domain could not be defined. In contrast to Akt1 and Akt3, we did not observe any interaction of Akt2 with DNA-PKcs. These findings are consistent with the functional studies performed demonstrating that knockdown of Akt1 and Akt3, but not of Akt2, result in impaired repair of radiation-induced DNA DSBs and increased cellular radiation sensitivity. Similarly to the interaction of Akt isoforms with DNA-PKcs, Akt1 and Akt3 knockdown, inhibited cell proliferation and clonogenic activity *in vitro* and tumor growth *in vivo.* This effect was not observed with Akt2.

Among three Akt isoforms, Akt1 interferes with DSBs repair mainly through NHEJ repair pathway.^6,8,10–12,15,20^ From our previous studies along with Park *et al.* demonstrated that the C-terminal domain of Akt1 interacts with DNA-PKcs.^8,9^ Here, we demonstrate that Akt1 mainly binds to the N-terminal domain of DNA-PKcs. It is known that a conformational change in the N-terminal domain of DNA-PKcs plays a critical role in enzymatic activity of DNA-PKcs.^21^ Thus, we suggest that the mechanism by which Akt1 activates DNA-PKcs in K-RAS-mutated cells involves binding to the N-terminal domain of DNA-PKcs, which stimulates DNA-PKcs kinase activity.^21^ Our data indicate that Akt3 binds to DNA-PKcs in a manner similar to that of Akt1. The Akt isoform-specific complex formation with DNA-PKcs may be due to the differences in the amino-acid sequences between different isoforms.^3^ Further studies will be necessary to identify the amino-acid sequences in the Akt isoforms that are crucial for the binding of Akt1 and Akt3, but not Akt2, to DNA-PKcs. In parallel to the activation of DNA-PKcs by Akt1, in the complex formed between Akt1 and DNA-PKcs,^11,12^ Akt is also activated by DNA-PKcs.^15,22^ Thus, complex formation of Akt1 and Akt3 with DNA-PKcs enhances the activation of Akt to a level that is not further elevated by irradiation. Likewise, enhanced Akt activity stimulates complex formation of both Akt1 and Akt3 with DNA-PKcs, and neither are stimulated by further radiation exposure (see Figures 2d and e and Supplementary Figure S1).

DNA-PKcs is the core enzyme for repair of DSBs through NHEJ and is involved in multiple tumor-associated pathways.^18^ DNA-PKcs-deficient cells are hypersensitive to IR.^23^ We previously reported that overexpression of mutated K-RAS(V12) in K-RAS wild-type cells results in enhanced radiation-induced DNA-PKcs dependent repair activity, which leads to cellular radioresistance.^17^ We now demonstrate that targeting the DNA-PKcs kinase activity reverses radioresistance of K-RAS-mutated A549 cells. Interestingly, the DNA-PKcs inhibitor (5 *μ*M) did not affect the Thr-2609 transphosphorylation of DNA-PKcs that is known to be regulated by ATM kinase.^24^ These data indicate that DNA-PKcs kinase activity in the absence of autophosphorylation at Thr-2609 can also play a significant role in the repair of radiation-induced DNA DSBs and radioresistance. The radiosensitizing effect achieved by the DNA-PKcs inhibitor was markedly stronger than the effect achieved by knockdown of Akt1 or Akt3 (Figure 5b and d). Together, our recent study and our previous report on the role of Akt1 in DNA-PKcs activity^8,10,11^ support the conclusion that the radiation-induced DNA-PKcs kinase activity is partially dependent on Akt (approximately 40–50%). On the basis of generating a strong radiosensitizing effect of the DNA-PKcs inhibitor, targeting DNA-PKcs is a much more effective strategy than targeting Akt1 or Akt3 for radiosensitization of solid tumors. However, because the PI3K/Akt pathway is one of the major survival pathways that is frequently upregulated in human tumors,^25,26^ Akt1 and Akt3 rather than DNA-PKcs are suggested to be tumor-specific targets as monotherapy as well as in combination with radiotherapy.

DNA-PKcs besides its role in NHEJ repair, functions as a transcription factor and regulates tumor-associated pathways and metabolism.^18^ In this study, we showed that Akt1 and Akt3 compared with Akt2 have opposite effects on cell proliferation and tumor growth of K-RAS-mutated cells. These differential effects may be because Akt1 and Akt3 bind to DNA-PKcs, but not Akt2. The data presented in Figure 6 support this conclusion. Compared with the data shown in Figure 6a, DNA-PKcs inhibitor, NT7441, significantly inhibited cell proliferation in cells expressing scr-shRNA as well as in cells expressing shRNA against different Akt isoforms. Interestingly, in DNA-PKcs inhibitor treated cells, Akt1-shRNA did not significantly inhibit cell proliferation. Likewise, DNA-PKcs inhibition completely abrogated the antiproliferative effect of Akt3-shRNA while DNA-PKcs inhibitors did not affect Akt2-shRNA. These data support the conclusion that the interaction of Akt1 and Akt3 with DNA-PKcs is crucial for the repair of radiation-induced DSBs and is a critical physiologic and functional interaction that regulates cell proliferation and tumor growth, especially in tumor cells with K-RAS mutation.

Together, DNA-PKcs physically interact with Akt1 as well as Akt3. This observation and the radiobiological data presented support the conclusion that targeting Akt1 and Akt3 isoforms in combination with radiotherapy may be effective in overcoming radioresistance of solid tumors with K-RAS mutations and an upregulated PI3K/Akt pathway.

## Materials and methods

### Antibodies and reagents

Antibodies against phospho-Akt, Akt1, Akt2, phospho-PRAS40, PRAS40, phospho-H2AX (Ser139) as well as the Akt inhibitor MK2206, Lipofectamine 2000, non-targeting siRNA, AKT1-siRNA, AKT2-siRNA VECTASHIELD Antifade Mounting Medium with DAPI, Alexa-647-labeled secondary antibody have been previously described.^7^ The anti-eGFP antibody (Cat. #3H9), anti-RFP antibody (Cat. #5F8) and GFP-Trap (Cat. #gta10) were kindly provided by ChromoTek (Martinsried, Germany). The DNA-PKcs inhibitor NU7441 (Cat. #S2638) were purchased from Selleck Chemicals (Munich, Germany). AKT3-siRNA (Cat. #M-003002–02) were purchased from Thermo Scientific Dharmacon (Bonn, Germany). Lipofectamine LTX reagent (Cat. #15338030) were purchased from Thermo Fisher Scientific (Ulm, Germany). Polyethylenimine (PEI) (Cat. 40,872-7) was purchased from Sigma-Aldrich (Taufkirchen, Germany).

### Cell lines

The established K-RAS-mutated NSCLC cell line A549 (ATCC, CCL-185) and breast cancer cell line MDA-MB-231 (ATCC, HTB-26) cells was used. A cell line authentication test was performed by Multiplexion (Immenstaad, Germany). HEK293T cells were used for the expression tests of recombinant DNA-PKcs constructs. A549 and HEK293T cells were cultured in DMEM medium routinely supplemented with 10% FCS and 1% penicillin–streptomycin and incubated in a humidified atmosphere with 93% air/7% CO_2_ at 37 °C. The cells were regularly tested for mycoplasma contamination.

We used shRNA to stably knockdown Akt isoforms in MDA-MB-231 cells. PLKO.1-puro vectors encoding either scrambled shRNA or shAKT1, shAKT2, shAKT3 were purchased from Sigma-Aldrich. The generation of pseudotyped lentiviruses and the measurement of transduction were performed as described previously.^27^ To generate stable knockdown cells, MDA-MB-231 cells were transduced with vectors containing scrambled shRNA or shAKT1, shAKT2 and shAKT3 and cells were selected following the addition of puromycin (Sigma-Aldrich) to the culture medium at a final concentration of 1.5 μg/ml for at least 1 week.

### DNA transfection

Transient transfection of A549 cells was carried out with Lipofectamine LTX according to the manufacturer’s guidelines. For transient transfection of HEK293T cells in p100 dishes, 24 μg DNA was mixed with 140 μl of a PEI solution (0.4 mg/ml in H_2_O, pH 7) that had been previously diluted in 600 μl DMEM to generate DNA/PEI complexes. After a 15-min incubation, this mixture was added to the cells.

### siRNA transfection, clonogenic assay and western blotting

The cells were transiently transfected with 50 nM siRNA against each of the Akt isoforms or the non-targeting siRNA using lipofectamine 2000 according to the manufacturer's instructions. The efficiency of the knockdown of the Akt isoforms after siRNA transfection was tested using western blotting as previously described.^28^ Forty-eight hours after the transfection, the cells were trypsinized and plated in six-well plates to evaluate the specific function of each Akt isoform in a post-irradiation clonogenic activity assay. After 24 h, the cells were irradiated with a single dose of irradiation (0, 1, 2, 3 or 4 Gy) and incubated for approximately 10 days. Thereafter, the colonies were stained with a solution of 0.05% w/v crystal violet. Colonies of >50 cells were scored as survivors. The clonogenic fraction of the irradiated cells was normalized to the plating efficiency of the unirradiated controls.^10^ To determine the radiosensitizing effect after the knockdown of each Akt isoform using specific siRNA, the DMF was calculated by dividing the irradiation dose that led to 37% survival in control-siRNA-transfected cells by the irradiation dose that led to 37% survival in Akt-siRNA-transfected cells. Similarly, radiosensitization by DNA-PKcs inhibitor NU7026 was calculated by dividing the irradiation dose that led to 37% survival in the vehicle (DMSO)-treated cells by the irradiation dose that led to 37% survival in DNA-PKcs inhibitor NU7026-treated cells. A DMF>1.00 indicates radiosensitization and a DMF<1.00 indicates radioprotection.

### Cloning of expression plasmids and plasmid preparation

AKT1, AKT2 and AKT3 were N-terminally fused to mCherry using the target backbone vector pEGFP-C1 Clonetech (Mountain View, CA, USA) in which EGFP has been replaced by mCherry. AKT-coding cDNA was amplified and *Xho*I/*Xba*I restriction sites were introduced by PCR using the following sets of oligonucleotides: AKT1-fwd 5′- AAA CTC GAG AAG GTG GAG GAG GTT CTA GCG ACG TGG CTA TTG-3′, AKT1-rev 5′- AAA TCT AGA TCA GGC CGT GCC GCT GGC CGA GTA GGA GAA C-3′, AKT2-fwd 5′- AAA CTC GAG AAG GTG GAG GAG GTT CTA ATG AGG TGT CTG TC-3′, AKT2-rev 5′- AAT CTA GAT CAC TCG CGG ATG CTG GCC GAG TAG GAG AAC-3′, AKT3-fwd 5′- AAA CTC GAG AAG GTG GAG GAG GTT CTA GCG ATG TTA CCA TTG-3′, AKT3-rev 5′- AAA TCT AGA TTA TTC TCG TCC ACT TGC AGA GTA GGA AAA TTG-3'. The PCR products were purified, digested with *Xho*I and *Xba*I and ligated into the target vector at the *Xho*I/*Xba*I restriction sites.

The DNA-PKcs constructs 1–426-N, 427–1400, 2401–3850 and 3700–4128-C were N-terminally fused to eGFP using the target backbone vector pEGFP-C1. DNA-PKcs-coding cDNA was amplified and *Hin*dIII/*Kpn*I restriction sites for DNA-PKcs-1-426-N or *Xho*I/*Kpn*I restriction sites for all other DNA-PKcs constructs were introduced by PCR using the following sets of oligonucleotides: DNA-PKcs-1-426-N-fwd 5′- AAA AGT CGA CGG TGG AGG AGG TTC TGC GGG CTC CGG AGC CGG-3′, DNA-PKcs-1-426-N-rev 5′- AAA AGG ATC CCT AAA CTG TGT CAA GGT ACA GCA AGA CGC, DNA-PKcs-427-1400-fwd 5′- AAA AGC TTT AGT TCC TGA GGT GTA TAC TCC-3′, DNA-PKcs-427-1400-rev 5′- AAG GTA CCC TAC ACA CAA ACA TCA GG-3′, DNA-PKcs-1401-2400-fwd 5′- AAC TCG AGT AAA TCT GAT GAA AGC TCT AAA G-3′, DNA-PKcs-1401-2400-rev 5′- AAG GTA CCC TAC ACC TCC AGA CAG AGT G-3′, DNA-PKcs-2401-3850-fwd 5′- AAC TCG AGT AGT ACT TTG TCG TGT GGA GG-3′, DNA-PKcs-2401-3850-rev 5′- AAG GTA CCC TAA TGT TTT CCT GAC ATT TTT G-3′, DNA-PKcs-3700-4128-C-fwd 5′- AAC TCG AGT AGA GAT TCC CGG TCA GTA TG-3′, DNA-PKcs-3700-4128-C-rev 5′- AAG AAT TCC TAC ATC CAG GGC TCC C-3′. The PCR products were purified and digested with *Hin*dIII/*Kpn*I or *Xho*I/*Kpn*I, respectively, and ligated into pEGFP-C1 at the respective restriction sites. The plasmids were prepared using the PureYield plasmid preparation system from Promega (Mannheim, Germany) according to the manufacturer’s protocol.

All generated constructs were confirmed by DNA sequencing (MWG, Martinsried, Germany). Protein expression of the generated constructs was tested by transfection of A549 cells using Lipofectamine LTX followed by microscopic analysis and by transfection of HEK293T cells using PEI followed by western blot analysis. Prior to western blot analysis, the eGFP-tagged DNA-PKcs domains were enriched by IP with GFP-Trap (ChromoTek GmbH) according to the manufacturer’s protocol.

### Immunoprecipitation

IP experiments were applied to analyze the interaction of eGFP-tagged domain(s) of the DNA-PKcs with mCherry-tagged Akt1, Akt2 or Akt3. To this end, the cells were co-transfected with plasmids expressing the eGFP-tagged DNA-PKcs fragments and mCherry-tagged Akt isoforms. The cells were lysed with lysis buffer^28^ 48 h after the transfection and subsequent experiment-dependent treatments. IP of the eGFP-tagged proteins was performed from the soluble fraction of the whole-cell lysates using GFP-Trap (ChromoTek) according to the manufacturer's instructions. Following the IP, the beads were washed with washing buffer (10 mM Tris/Cl, 150 mM NaCl, 0.5 mM EDTA, phosphatase inhibitors and protease inhibitor cocktail) and suspended in SDS sample buffer. After dissociating the immune complexes from the beads (10 min at 95 °C), the beads were collected by centrifugation and the supernatant was subjected to SDS-PAGE. Co-precipitation of Akt isoforms was tested using isoform-specific antibodies.

### γH2AX foci assay

An analysis of the γH2AX foci was used to evaluate the repair of the IR-induced DSBs following siRNA-mediated knockdown of the Akt isoforms. The cells were transfected with 50 nM siRNA against the Akt isoforms or with control non-targeting-siRNA using lipofectamine. Forty-eight hours after this transfection, the cells were irradiated with 4 Gy and were subsequently fixed using PBS/2% formaldehyde at 24 h post-irradiation. The cells were stained with the antibody to phospho-H2AX (Ser139) as previously described.^10^ The numbers of γH2AX foci per cell were counted, and the averages were graphed using the Simplot graphics software.

### Proliferation assay

To test the effect of Akt isoforms on cell proliferation, MDA-MB-231 cells (3×10^4^) expressing scramble-shRNA, AKT1-shRNA, AKT2-shRNA and AKT3-shRNA were used. Cells were seeded in 6 cm tissue culture plates and let to grow. At days 4, 5, 6 and 7 after seeding, cells were collected by trypsinization, counted and growth curves were prepared. The cell PDT was calculated. Statistical analysis was performed to compare PDT in cells expressing scramble-shRNA with the cells expressing shRNA against either of Akt isoforms. In a further experiment, MDA-MB-231 cells (3×10^4^) expressing scramble-shRNA, AKT1-shRNA, AKT2-shRNA and AKT3-shRNA were split and treated with DNA-PKcs inhibitor NU7441 (10 *μ*M) after 24 h. On day 6 after treatment cells were trypsinized and counted.

### Mouse xenografts tumor growth assay

Athymic nude mice (4- to 6-week-old females) were obtained from Harlan Laboratories (Indianapolis, IN, USA). All animal procedures and maintenance were conducted in accordance with the institutional guidelines of the University of Wisconsin. Mice were randomized and injected with MDA-MB-231 cells (2×10^6^ cells) stably transfected with scramble-shRNA, AKT1-shRNA, AKT2-shRNA or AKT3-shRNA in both dorsal flank. Tumor volume measurements were evaluated by digital calipers and calculated by the formula (*π*/6 x (small diameter)^2^×large diameter) weekly from day 7 after tumor cells injection.

### Statistics and densitometry

Student’s *t*-test was used to evaluate the significance of difference between two groups of data. *P*-values less than 0.05 were considered statistically significant (*P<0.05; **P<0.01; ***P<0.001). Densitometric quantification analyses of the immunoblots was performed using the ImageJ 1.44p software (http://imagej.nih.gov/ij/) and Image Studio Light Ver 5.2.

## Additional information

**Publisher’s note:** Springer Nature remains neutral with regard to jurisdictional claims in published maps and institutional affiliations.
