# Supplementary material for: Akt1 and Akt3 but not Akt2 through interaction with DNA-PKcs stimulate proliferation and post-irradiation cell survival of K-RAS-mutated cancer cells
Source: Cell Death Discov. 2017 Oct 30;3:17072–. doi: 10.1038/cddiscovery.2017.72 (PMC5661268; doi:10.1038/cddiscovery.2017.72)
Supplement: Supplementary Figures [file cddiscovery201772-s1.pptx]

## Slide 1
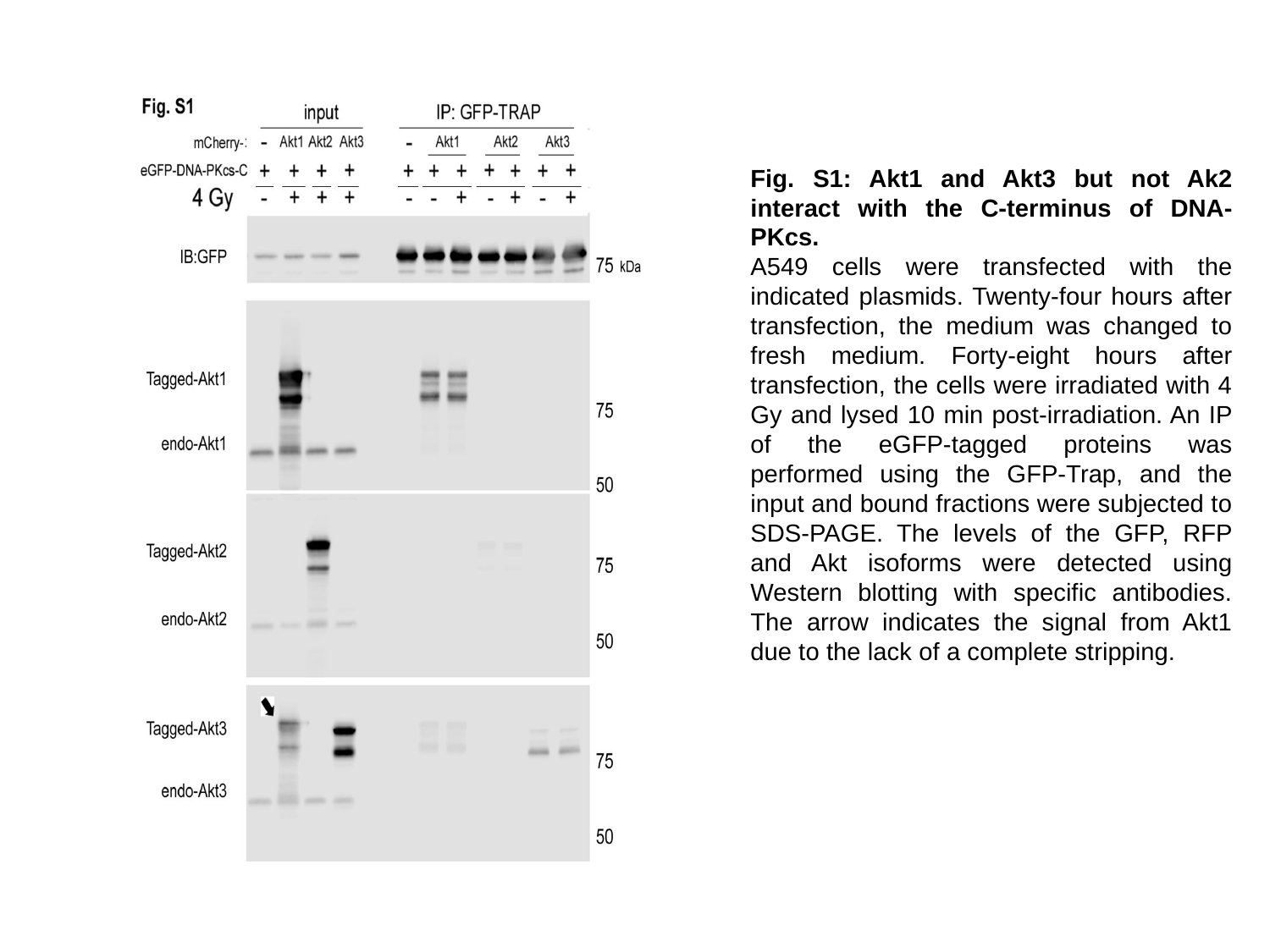

Fig. S1: Akt1 and Akt3 but not Ak2 interact with the C-terminus of DNA-PKcs.
A549 cells were transfected with the indicated plasmids. Twenty-four hours after transfection, the medium was changed to fresh medium. Forty-eight hours after transfection, the cells were irradiated with 4 Gy and lysed 10 min post-irradiation. An IP of the eGFP-tagged proteins was performed using the GFP-Trap, and the input and bound fractions were subjected to SDS-PAGE. The levels of the GFP, RFP and Akt isoforms were detected using Western blotting with specific antibodies. The arrow indicates the signal from Akt1 due to the lack of a complete stripping.

## Slide 2
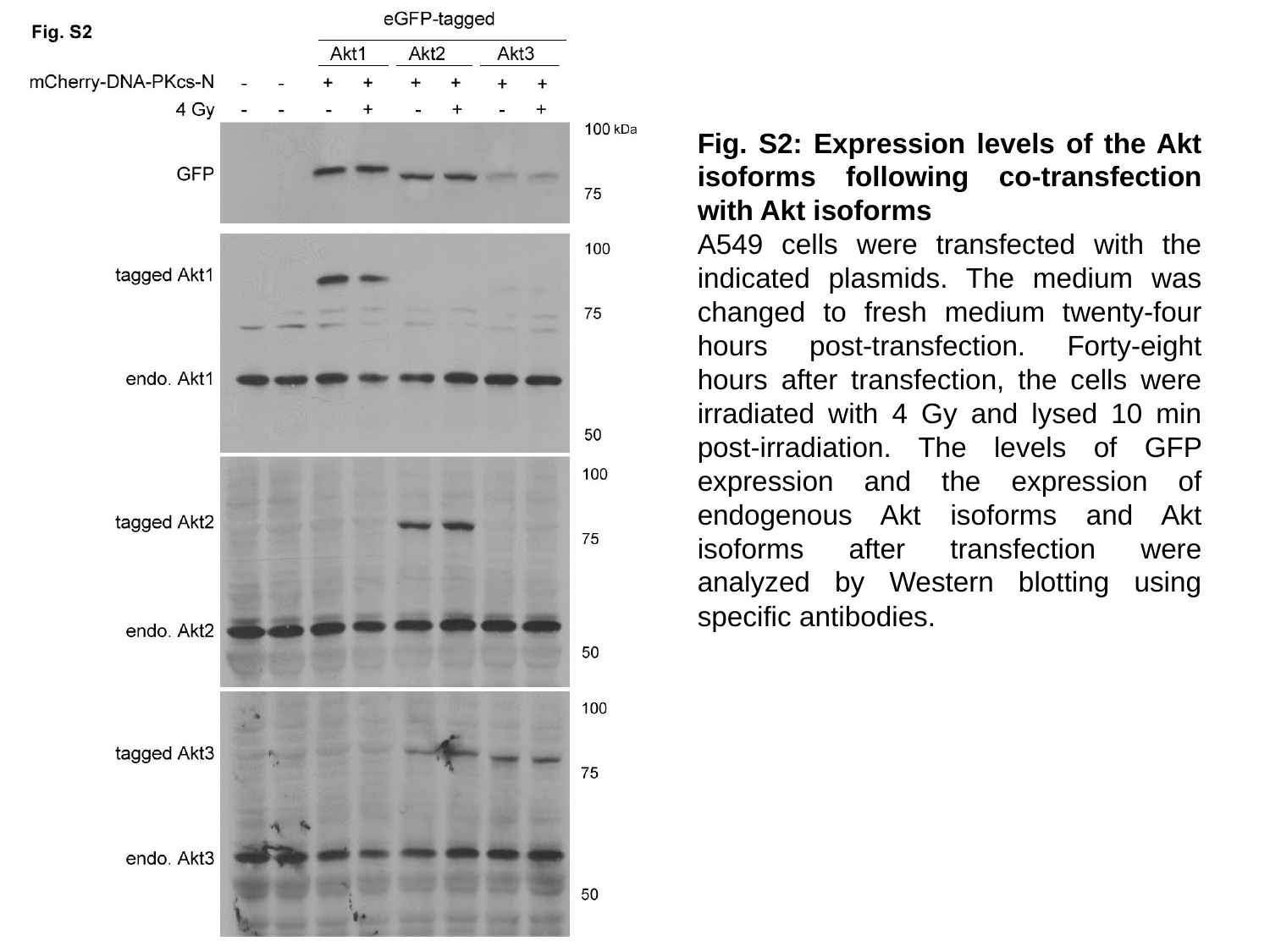

Fig. S2: Expression levels of the Akt isoforms following co-transfection with Akt isoforms
A549 cells were transfected with the indicated plasmids. The medium was changed to fresh medium twenty-four hours post-transfection. Forty-eight hours after transfection, the cells were irradiated with 4 Gy and lysed 10 min post-irradiation. The levels of GFP expression and the expression of endogenous Akt isoforms and Akt isoforms after transfection were analyzed by Western blotting using specific antibodies.

## Slide 3
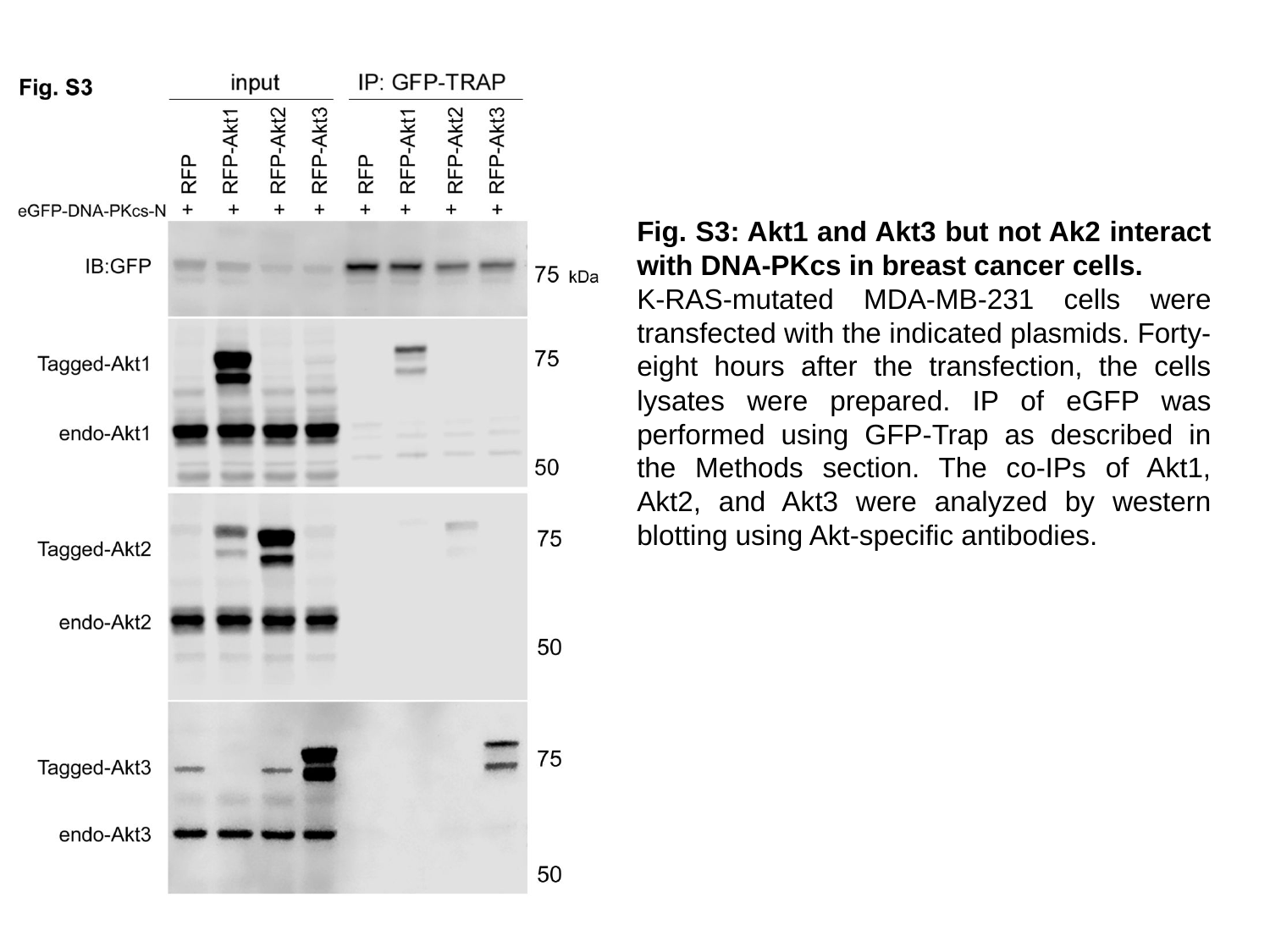

Fig. S3: Akt1 and Akt3 but not Ak2 interact with DNA-PKcs in breast cancer cells.
K-RAS-mutated MDA-MB-231 cells were transfected with the indicated plasmids. Forty-eight hours after the transfection, the cells lysates were prepared. IP of eGFP was performed using GFP-Trap as described in the Methods section. The co-IPs of Akt1, Akt2, and Akt3 were analyzed by western blotting using Akt-specific antibodies.
